# Dr. James Samuel W. Williams

**Published:** 1913-09

**Authors:** 


Dr. James Samuel W. Williams, Victoria College, 1867, died at his home, Oakville, Ont., June 4, aged 72.

